# Treatment of Disabled Sailors and Soldiers

**Published:** 1918-02-02

**Authors:** 


### THE VOLUNTARY HOSPITALS.

### Treatment of Disabled Sailors and Soldiers.

We have received the following important statement which we deal with fully in a leading article :

The Conference convened by the British Hospitals Association, and held at Westminster Hospital on October 19, 1917, referred to the Council of the Association the consideration of the question of the treatment of War Pensioners, and in view of the "Instructions and Notes on the Treatment of Disabled Men" issued by the Ministry of Pensions, authorised the Council to negotiate terms with the Ministry of Pensions.

Representatives of the Governors and Staffs of certain large London Hospitals had met and considered this question, and passed some resolutions which were sent to the British Hospitals Association.

At a meeting of the Council held on December 14, 1917, certain resolutions were agreed, and arrangements made for a Deputation to the Minister of Pensions, which was received on December 20, when the proposals made on behalf of the Hospitals were considered.

As a result of the discussion between the deputation and the Ministry of Pensions, it was decided that the consideration of certain details should be referred to a Joint Committee of the British Hospitals Association and of the Ministry.

Two meetings of the Joint Committee were held, with the result that the following arrangements were agreed to. These have now been adopted by the Minister of Pensions.

The Ministry are only empowered to provide treatment for men disabled by reason of service in the present war, and for these men only so far as their disablement, or any incapacity connected with it, necessitates medical attention after discharge.

J. Courtney Buchanan,
Conrad W. Thies,
January 24, 1918. Hon Secretaries.

*Memorandum of the Arrangements agreed by the Joint Committee of the British Hospitals Association and the Ministry of Pensions, for the treatment of Disabled Sailors and Soldiers, sent to Civil Hospitals by, or on behalf of, the Ministry of Pensions.*

1. That the Voluntary Hospitals which undertake the work will examine and, if found suitable, treat all Disabled Sailors and Soldiers sent to them by any Local War Pensions Committee on the recommendation of a medical Referee or a Medical Board, or other Medical Officer appointed by the Ministry of Pensions.

It will rest with the authorities of the Hospital to decide, after examination of the man, whether they can accept a patient for treatment and whether he should be treated as an In- or Out-patient.

2. It is understood that if there is any difference of opinion as to the suitability of the case for treatment in the Hospital, from whatever cause this disagreement may arise, the Hospital Authority or Medical Officer of the Hospital will communicate at once with the Medical Referee, with the Local War Pensions Committee, or with the Medical Officer of the Ministry of Pensions who may have sent the case to the Hospital, stating the grounds of objection and suggesting also the treament advised.

3. *In-patient Treatment.*—That a minimum of 5s. per day will be paid by the Ministry of Pensions for all such cases as are admitted for In-patient treatment on the understanding that in the case of any Hospital which can show on good evidence that the cost of treatment is reasonably higher in that Hospital a higher charge may be agreed with the Ministry of Pensions up to a maximum of 7s. per day.

*Note.*—It was agreed on this point that Hospitals which have no resident staff will not be allowed more than 5s. per day. Only Hospitals which are fully equipped, and contain Bacteriological, Massage, *x*-ray and other special departments, will be eligible for the maximum rate of 7s. per day on the basis of the actual evidence produced in support of their claim.

4. *Out-patient Treatment.*—That in the case of Hospitals which are paid a flat rate of 5s. for In-patient treatment, the normal rate for Out-patients shall be 1s. per attendance. In those Hospitals in which the flat rate for In-patients is 6s. or over, the rate for Out-patients shall be 2s. per attendance.

*Note.*—The foregoing terms in Clauses 3 and 4 do not preclude any arrangement by which the cost of the treatment is defrayed at a composition rate per case, in place of payment per day, for In-patients, or per attendance for Out-patients, if such arrangement should be more convenient.

5. That these charges as agreed to by the Ministry of Pensions be made to the Hospitals, and that they cover all costs including any allowances made by the Hospital in recognition of Medical Services.

6. That the Authorities of the Hospital undertake to keep an accurate record of the attendance and treatment of disabled men, to notify the Local War Pensions Committee of the admission and discharge and Out-patient attendance of all patients sent by them or by the Ministry of Pensions, and to furnish a certificate at the conclusion of treatment, stating on medical opinion :

(1) The general condition of the patient;
(2) Whether or not he needs further treatment which the Hospital is not in a position to give; and, if so, what kind of treatment, *e.g.*, Convalescent.

The foregoing notifications and certificates to be covered by the inclusive capitation charge made for the attendance and treatment of patients.

7. In cases where the Ministry of Pensions ask for a detailed Specialist Report, whether on Form B. 179 or otherwise, a fee of 21s. will be paid. It is understood that neither the Medical Referees nor the Local War Pensions Committees are entitled to call for such a Report from Hospitals; but that if in any case such Report is considered necessary, the Local Committee, having first consulted the Medical Referee, must obtain the authority of the Ministry of Pensions.

In future, where the Hospital is asked for a Report by a Local Committee without such authority, the request should be returned to the Committee with an intimation that the case should be sent, in the first instance, to the Medical Referee. It should not be necessary for Local Committees to require the kind of detailed Report for which the special fee in question is payable; but it will be considered whether there are any classes of case for which such Reports might properly be required by them.

8. Where a disabled man is admitted for treatment as an emergency case without authority from the Local War Pensions Committee, or the Ministry of Pensions, the Hospital shall communicate without delay with the Local Committee, stating the disease for which treatment is required, with a view to ascertaining whether the case is one in which the charges in paragraphs 3 and 4 above can be made; and where in such case responsibility is

(Continued on p. iv.)

